# Design and Fabrication of a Novel Transplant Combined with Human Bone Marrow Mesenchymal Stem Cells and Platelet-rich Fibrin: New Horizons for Periodontal Tissue Regeneration after Dental Trauma

**Published:** 2017

**Authors:** Hamid Moradian, Azade Rafiee, Maryam Ayatollahi

**Affiliations:** a *Department of Pediatric Dentistry, School of Dentistry, Shiraz University of Medical Sciences, Shiraz, Iran.*; b *Bone and Joint Diseases Research Center, Shiraz University of Medical Sciences, Shiraz, Iran.*; c *Transplant Research Center, Shiraz University of Medical Sciences, Shiraz, Iran.*

**Keywords:** Tissue engineering, Biopharmaceuticals, Mesenchymal stem cells, Platelet-rich fibrin, Cell delivery

## Abstract

Avulsed teeth that are replanted dried are more prone to loss. Recent tissue engineering studies focus on fabrication of various cell delivery systems to improve the overall prognosis of such teeth. To evaluate this new cell transplant method, we initially aimed at designing of PRF scaffold and determining BMMSCs viability and function on the fabricated scaffold. To test this concept *in-vitro*, human BMMSCs were isolated and characterized by cell surface marker, and their potential on osteogenic/adipogenic differentiation. The biological effects of PRF scaffold on human BMMSCs were then investigated by cell proliferation assay. The data were quantified for statistical analysis. It was found that PRF significantly induced BMMSCs proliferation throughout the incubation period due to its growth factor secretion. Results of MTT assay showed an increasing trend during the testing period from day 1 to day 7. The result of scanning electron microscopy showed that BMMSCs could tightly adhere to fibrin scaffold just shortly after seeding. These data suggest that the BMMSCs/PRF construct has the potential to improve the clinical prognosis of replanted avulsed teeth in future. Additional studies are needed to be performed before its clinical use.

## Introduction

Tooth avulsion, one of the most severe forms of dental trauma, is defined as the complete displacement of tooth out of its alveolar socket (1). Replantation is widely accepted as a standard treatment option for avulsion supposed that the avulsed tooth is immediately replanted within an hour and during the extra-alveolar time the tooth is preserved in an appropriate medium such as milk or physiologic saline. However, it is not always possible to replant the tooth immediately. This causes desiccation of root surface and leads to loss of vitality of cells in the remaining periodontal ligament (PDL), hence, results in rapid root resorption, ankylosis, and finally tooth loss (2).

An increased interest has risen to manage this condition more effectively (3). Many researchers have investigated the effects of various storage media on PDL healing prior to replantation (4). Others have tried to improve the healing of periodontal tissue by application of different substances such as recombinant growth factor (5), enamel matrix derivatives (Emdogain^®^), and antibiotics like tetracycline (6). However, these treatment modalities did not significantly improve the viability and proliferation of PDL cells (7). To deliver and sustain these therapeutic substances on the root surface, constructing a specific scaffold is essential. It is obvious that conventional transplants cannot be implanted in the narrow space between alveolar bone and cementum that ranges from 0.15 to 0.38 mm in width (8). Different cell-sheet delivery systems have been developed to adequately deliver PDLSCs such as endothelial cells, hepatocytes, macrophages, and retinal pigmented cells (9). Platelet activated with thrombin such as platelet-rich plasma (PRP) has also showed tissue healing due to continuous delivery of various growth factors and proteins needed for wound healing (10). Platelet-rich fibrin (PRF), the second generation platelet concentrate, lacks biochemical blood handling and it, therefore, has definite advantages over traditionally prepared constructs (8). PRF clot forms a strong natural fibrin matrix full of growth factors with a three-dimensional (3D) structure which can stimulate proliferation of PDL cells, gingival fibroblasts, and osteoblasts (11). Bone marrow mesenchymal stem cells (BMMSCs) are a well-characterized population of adult stem cells that might be precursors of different mesenchymal cells such as chondrocytes, osteoblasts, adipocytes, and myoblasts (12). These cells express various adhesion molecules such as CD29, CD44, CD90, CD105, CD166, SH2, SH3, and SH4 but do not express CD34 since they have been phenotypically specified as non-hematopoietic stem cells (13). PDLSCs and BMMSCs have many similar characteristics in common. Both cells express early mesenchymal stem cell surface markers (14). Besides, both cells express the same surface markers of bony tissues. These results suggest BMSCs’ potential to develop periodontal ligament (15)

It is reported that BMMSCs can communicate with dental tissues (16). Our previous findings suggest that BMMSCs can be able to transdifferentiate into multi-lineage tissues in response to specific culture conditions (17). BMMSCs also seem to differentiate into specific periodontal tissue cells such as cementoblasts, osteoblasts and PDL fibroblasts, with an unknown mechanism when placed in periodontal environment (18).

There are only a few *in-vitro *studies which have evaluated the effects of PRF construct on MSCs proliferation, function, and differentiation (3, 19). Based on our searches, there was only one published study regarding the effect of BMMSCs cultured on PRF for root regeneration (20). To the best of our knowledge, none of these studies have evaluated BMMSCs on PRF with the aim of periodontal regeneration. To evaluate this new cell transplant method, this study aimed to design PRF scaffold and determine BMMSCs viability and function on the fabricated scaffold.

## Experimental

*Isolation and characterization of BMMSCs*

This experimental study was performed in Transplant Research Center, Shiraz University of Medical Sciences, Shiraz, Iran. Human MSCs were obtained from 5 mL bone marrow aspirates from iliac crest of normal donors within the age range of 19-45 years. They were donors of bone marrow to a related patient after obtaining approval of Ethic Committee of Shiraz University of Medical Sciences. Written informed consents were also obtained to allow analysis of the clinical data mentioned in this study. Each sample of aspirate was diluted 1:1 with Dulbecco’s modified Eagle’s medium (DMEM)-low glucose (1,000 mg/L) (Invitrogen, Merelbeke, Belgium) contained 10% fetal calf serum (Invitrogen, Merelbeke, Belgium), and supplements. The isolation method was according to our previously reported method (21). The cells layered over about 5 mL of ficoll (Lymphoprep; Oslo, Norway), then centrifuged at 338×g for 15 min to obtain mononuclear cell (MNC) layer. The MNC layer was seeded on culture flasks, and maintained at 37 °C in 5% CO_2_ atmosphere. In order to obtain MSCs cells, the adhered monolayer cells were detached and expand for successive passages. At each passage, the cells were analyzed for viability, and characterized by flow cytometric analysis, differentiation to osteocytes and adipocytes. Each experiment described in here was replicated for three times.

*Flow cytometric analysis of cell surface markers*

After digesting with 0.25% trypsin (Gibco, USA) and washing with PBS (Gibco, USA) for three times, the cells were adjusted to a concentration of 1 × 10^6^ cells/mL. Then, the cell surface antibodies were added to the microtubes according to the manufacturer’s instructions. These antibodies were CD90 (eBioscience, USA), CD34 (eBioscience, USA), CD45 (eBioscience, USA), CD73 (eBioscience, USA). An isotype control with FITC-labeled was included in each experiment. Negative control included non-stained cells and isotype-control stained cells. The labeled cells were thoroughly washed with PBS and analyzed on a flow cytometer (FACS Calibur Becton, Dickinson, USA), using WinMidi software (Scripps Research Institute; San Diego, USA).

*Osteogenic/adipogenic differentiation of MSCs*

The potential of the isolated cells to differentiate into osteogenic and adipogenic lineages was examined. For osteogenic differentiation, the 3^rd^-passage cells were treated with osteogenic medium for 4 weeks. Osteogenic medium consisted of DMEM supplemented with 10^-8^ M/L dexamethasone (Sigma-Aldrich, St. Louis, USA), 10 mM/L glycerol phosphate (Sigma-Aldrich, St. Louis, USA), 3.7 g/L sodium bicarbonate (Sigma-Aldrich, St. Louis, USA), and 0.05 g/L ascorbic acid (Sigma-Aldrich, St. Louis, USA). Osteogenesis was assessed by alizarin red staining. To induce adipogenic differentiation, the 3^rd^-passage cells were treated with adipogenic medium for 2-3 weeks. Adipogenic medium consisted of DMEM supplemented with 1 M/L hydrocortisone (Sigma-Aldrich, St. Louis, USA), 0.05 g/L ascorbic acid, 0.05 g/L indomethacin (Sigma-Aldrich, St. Louis, USA), and 10^-6^ M/L dexamethasone. The mineral nodules and lipid areas were observed using a phase-contrast microscope and the images were captured.

*Preparation of cell construct*

After filling the written consent, blood sample of each volunteer participant was taken from the median cubital vein and transferred into a 10 mL glass tube. The tube was centrifuged at 400×g for 10 min. After the fibrin clot was obtained and compressed, the fibrin membrane was then cut into small pieces.

*Cell proliferation assay*

Cell proliferation was assessed using Thiazolyl Blue Tetrazolium bromide (MTT) (Sigma-Aldrich Co., St. Louis, MO, USA) assay. Metabolically active cells reduce MTT tetrazolium salt to formazan. At different culture times (1, 5, 7, 9, and 12 days), cell seeded construct group, and cell control group (without scaffold) (2 × 10^5^ cell/mL in each group) were incubated in MTT solution (0.5 mg/mL, 37 °C/5% CO_2_) for 3 h. The intense red colored formazan derivatives formed were dissolved and the absorbance was measured at 570 nm and 630 nm as reference wave length, by an absorbance microplate reader (BioTek Instruments, Inc., USA). The recorded optical density (OD) values of different wells were taken and used to determine the final average reading. The growth curves for the MSCs cultured in all groups were illustrated, and the results from different groups were statistically compared. 

*Statistical analysis*

All *in-vitro* experiments were performed independently for each cell voluntaries and repeated at least three times. The results were expressed as the mean ± standard deviation and were compared using student’s *t*-test. A level of *p *< 0.05 was accepted as being statistically significant. The analyses were performed using the IBM SPSS 17.0 software (SPSS, USA).

## Results

*Isolation and identification of MSCs*

**Figure 1:**
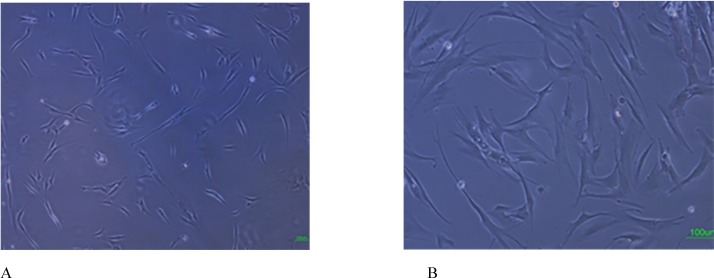
Isolation and culture of human bone marrow derived MSCs. A: Adherent monolayer was achieved in the following 6-7 days; B: As the culture proceeded, the cells were both small spindle, and wide-shaped morphology. Scale bar for Figures A-B: 100 µm

**Figure 2:**
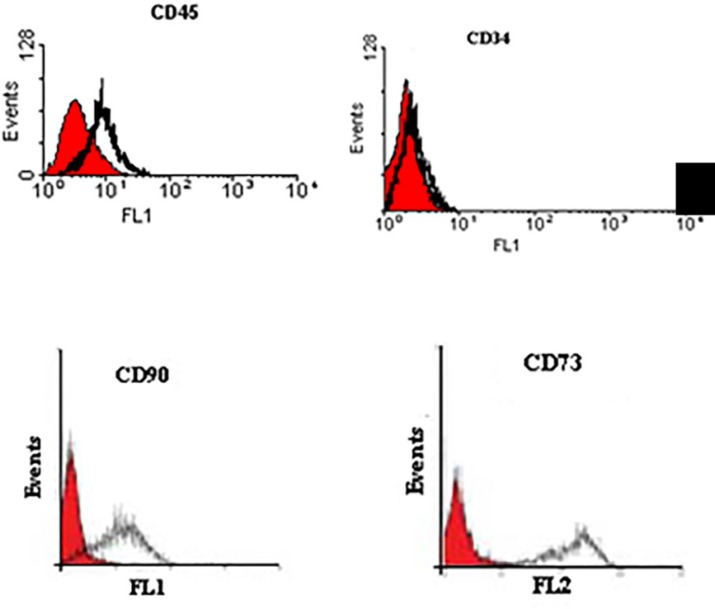
Immuno-phenotyping of human bone marrow derived MSCs using ﬂow cytometry. The surface phenotypic markers were positive for CD90, and CD73. Additionally, no cells expressed the hematopoietic markers CD45, CD34. The shaded area shows the profile of the negative control

**Figure 3:**
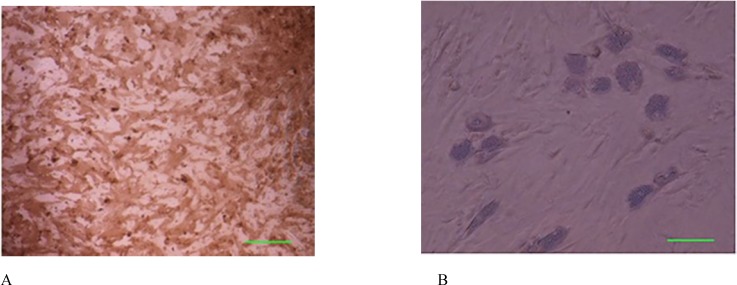
Osteogenic and adipogenic differentiation of bone marrow mesenchymal stem cells in the third passage. (A): Osteogenic differentiation was positive for alizarin red staining. (B): The adipose droplet in differentiated cells after incubating with adipogenic media. Scale bar for Figures A-B: 50 µm

Cells successfully grew from bone marrow aspirate sample after 6-8 days of culture. The cells demonstrated a spindle-shaped morphology (Figure 1A). The fibroblast-like cells which were rapidly grown and showed homogeneous morphology were selected for culture expansion (Figure 1B). Trypan blue staining analysis showed viability between 98% and 100% in human MSCs. Their surface phenotype was determined by ﬂow cytometry, and was positive for CD90, and CD73. Additionally, no cells expressed the hematopoietic markers CD45, CD34 (Figure 2). The capacity for multiple-directional differentiation is also one of the key properties of any MSC line. Hence, the MSCs were induced into osteogenic and adipogenic media to evaluate their differentiation potentials. After 4 weeks of osteogenic induction, Alizarin Red-positive mineral deposits could be observed indicating the osteogenic potential of cells (Figure 3A). Meanwhile, the lipid vacuoles eventually combined and filled the cells. The accumulation of lipid in these vacuoles was assessed histologically (Figure 3B). These results suggested that MSCs were successfully obtained from the human bone marrow aspirate.

**Figure 4:**
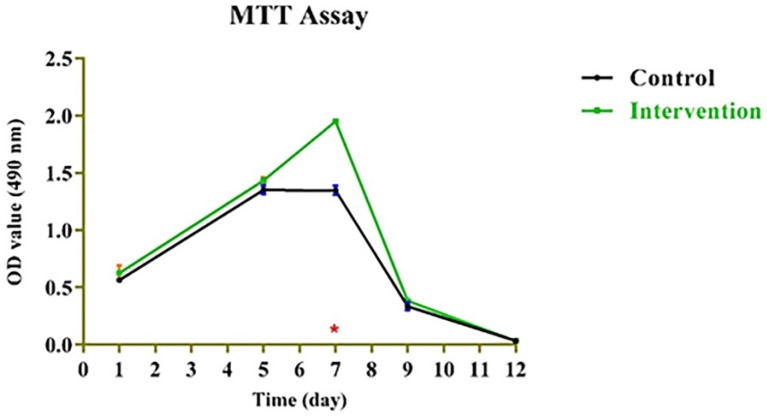
Mitochondrial redox activity of bone marrow mesenchymal stem cells on platelet-rich fibrin.

**Figure 5.**
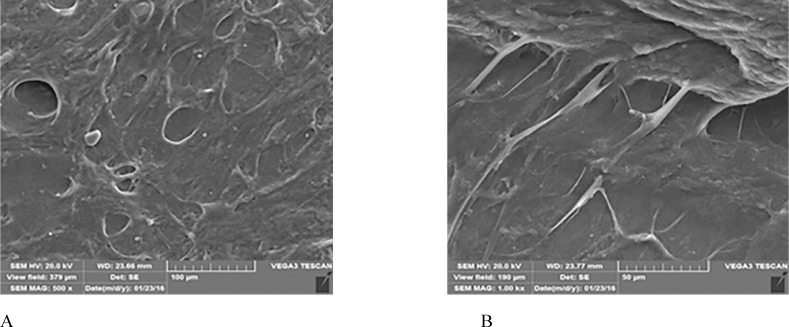
The morphology of the BMMSCs adhering on PRF surface at day 5 obtained by scanning electron microscopy. A: Porous shape of PRF scaffold. B: Cells extended a large number of cell processes into the pores of the PRF fibrins

**Table 1:** EC50 values for bone marrow mesenchymal stem cells (BMMSCs) fixed on platelet rich fibrin (PRF) during 1-12 day incubation period based on the dose-response curves as derived from the MTT assay. Data presented as EC50 values (*n *= 3) ± SD; SD: Standard deviation

**Day**	**Mean (control) ± SD**	**Mean (intervention) ± SD**	***p*** **-value**	**Significance**
1	0.564 ± 0.0001	0.626 ± 0.0044	0.186679	-
5	1.351 ± 0.0008	1.434 ± 0.0008	0.0658778	-
7	1.346 ± 0.0017	1.950 ± 0.0	1.431855e-005	Yes
9	0.335 ± 0.0012	0.381 ± 0.0001	0.884319	-
12	0.034 ± 0.0	0.034 ± 0.0	1.0	-

*Assessment of cell proliferation on PRF scaffold*

Mitochondrial redox activity of human BMMSCs on PRF was evaluated by reduction of MTT by mitochondrial succinate dehydrogenase to yield formazan crystals. After 3-4 h of cell incubation with MTT, formazan crystals were developed in living cells. The intense red colored formazan derivatives were dissolved and the absorbance was measured. Results presented in Figure 4 show that MTT-reducing activity was significantly higher in the cells that were cultured on PRF (*p < *0.05) compared to those cultured on control throughout the 12-day incubation period. Obtained values for bone marrow mesenchymal stem cells (BMMSCs) fixed on platelet rich fibrin (PRF) during 1-12-day incubation period based on the dose-response curves as derived from the MTT assay were presented in Table 1.

However, no dose-dependent effect was found among the PRF-containing groups except at day 7 (Figure 4). As for the MTT assay, the absorbance values for the growth curves in all groups increased during the testing period from day 1 to day 7.

*Ultrastructure of the MSCs/PRF construct*

The ultrastructure of the MSCs/PRF construct, observed by SEM, indicated that the cells secreted extracellular matrix, expanded well and extended multiple cell processes into the pores of the PRF fibrins. The cells formed an integral biomaterial with PRF (Figures 5A-B).

## Discussion

It is not always possible to replant the avulsed tooth immediately. Desiccation of root surface leads to loss of vitality of cells in the remaining periodontal ligament (PDL), which consequently results in rapid root resorption, ankylosis and eventually tooth loss (2). To better manage this condition, many researchers have tried to improve PDL vitality by various storage media (4) or by application of different substances such as recombinant growth factor (5), enamel matrix derivatives (Emdogain^®^) and antibiotics like tetracycline (6) before replantation procedure. However, these treatment modalities did not significantly fulfill the clinical expectations (7). Knowing that the major cause of tooth replantation failure is the damaged PDL cells, it would be logical to suppose that the adequate cells delivery system may improve the long term prognosis of such teeth (3). Since PDL space ranges from 0.15 to 0.38 mm (8), we have cultured human BMMSCs on PRF to create a cell construct and we believe that this delivery system can effectively sustain stem cells on the scaffold and root surface. We have also evaluated the viability and function of these mesenchymal stem cells on the prepared scaffold. 

PRF can improve both soft and hard tissue healing. The noticeable advantages over platelet-rich plasma (PRP) are ease of application, low cost, without the need for biochemical modification. Besides, PRF forms an elastic fibrin mesh, which favors various cytokine entrapment and preserves the growth factors from proteolysis (11). In our study, BMMSC proliferation capacity was stimulated by PRF. This result is consistent with other researches (19, 22) which might be due to high levels of growth factors entrapped in PRF scaffold (8). Based on the MTT assay results of the present study, the number of BMMSCs in the intervention group increased significantly (*p *< 0.05) than that in the control group until the 7^th^ day of incubation. This result was similar to results yielded by the study of Zhao *et al.* (3), though, they used PDLSCs instead of BMMSCs and considered a 7-day incubation period for their MTT assay. We evaluated cell proliferation until the 12^th^ day. As demonstrated in Figure 4, the absorbance values of both groups showed an increase until the day 7 with significant difference between the intervention and control group, and then gradually started to decrease. This is consistent with the growth curve of BMMSC evaluated in an *in-vitro* study by Peng *et al.* (23). One reason to this decrease might be lower O_2 _tension in small culture wells which is produced during cell cultivation and can instigate cell death. According to Lu *et al.* study, the rapid disappearance of infused MSCs does not advocate the absence of functional effect in these cells (24). In fact, MSCs secrete soluble factors rapidly and stimulate responses by surrounding cells only by their presence. We found that BMMSCs could tightly adhere to fibrin scaffold just shortly after seeding. Cell/scaffold construct ultrastructure observed by SEM in the day 5, indicated that extra-cellular matrix secretion was activated and cells’ pseudopodia penetrated in to the scaffold to produce a 3D structure (Figure 5). These characteristics make PRF an appropriate cell delivery vehicle in periodontal regeneration.

Tissue engineering approaches use bio-compatible and bio-degradable scaffolds in combination with appropriate cells. We have previously reported the effect of allogeneic cultured BMMSC on chitosan scaffold in regeneration and function of injured tissues (22). Various studies have used autologous cultured BMMSCs with atelocollagen, collagen scaffold, pluronic F127, PRP and microcarrier gelatin beads vehicle as biomaterial-based cell-delivery strategies (25-29). PDLSCs have the potential to regenerate the periodontal tissue (15). Hasegawa *et al.* labeled BMMSCs with green fluorescent protein (GFP) and the labeled cells were detectable four weeks after transplantation by immunohistochemistry assay. The periodontal defect was regenerated with periodontal tissue. Cementoblasts, osteoblasts, osteocytes and fibroblasts of the regenerated tissue were positive for GFP which means that BMMSCs could differentiate to various periodontal tissue cells (30). BMMSCs have the potential to regenerate various tissues including periodontal tissue in response to stimulation, provoked by multiple environmental factors (31). PDLSCs and BMMSCs have many similar characteristics in common. Both cells express early mesenchymal stem cell surface markers and the same surface markers of bony tissues (14). These results suggest BMSCs’ potential to develop periodontal ligament. Moreover, access to PDLSCs needs tooth extraction and because of the small sample size and rarity of these cells, the number of recoverable PDLSCs is limited. On the contrary, BMMSCs can be achieved not only easier but also in a considerable larger numbers (12). Zhang *et al.* reported that the BMMSCs might show the stronger immunomodulation in local microenvironment compared to PDLSCs (15). 

There are some challenges that need to be dealt with before the clinical use of BMMSCs based on PRF scaffold. First of all, we need to develop a protocol for clinical scale of production which fulfills cell survival and dividing capacity (30). BMMSCs do not express co-stimulatory antigens so they do not provoke humoral immune response when used as allogeneic stem cell based therapeutics (32). It seems that various animal studies are needed to determine their long term safety for periodontal repair (33). Use of *in-vitro* cultured stem cells for delayed-replanted avulsed teeth seems to be difficult for clinical application because this methodology is time-consuming and for the traumatized tooth, a delay of few weeks for cell culture is not permitted (3). The first study to use cell/scaffold construct for periodontal regeneration in human was performed by Feng *et al.* in 2010 (34), proposed that this method would clinically be translatable. Autologous stem cell transplantation (SCT) has been broadly accepted as a robust treatment method for many diseases, such as hematological malignancies (35, 36). Moreover, Holoclar^®^, an autologous limbal stem cell therapy for repairing damaged cornea, has achieved a conditional marketing approval by European Commission as the first stem cell therapy based biopharmaceuticals in 2015 (37). Therefore, close cooperation between tissue engineers, pharmaceutical biotechnologists and dentists might help to overcome this dilemma by reducing the time for a successful use of this procedure as an effective clinical treatment modality. Moreover, this *in-vitro* methodology may introduce a potential tissue engineering-based treatment option for saving more avulsed teeth in near feature if sufficient innocuous cells are readily available on demand.

## Conclusions

Our data suggest that the use of cell sheet fragments of BMMSCs and PRF scaffold is effective. The PRF growth factors may improve the overall prognosis of dry-stored avulsed teeth. In general, the use of this method may shed some light on the treatment of avulsed teeth in future dental practice; however, additional studies are needed to be performed before its clinical use.

## References

[1] Martin MP, Pileggi R (2004). A quantitative analysis of Propolis: A promising new storage media following avulsion. Dent. Traumatol..

[2] Flores MT, Andersson L, Andreasen JO, Bakland LK, Malmgren B, Barnett F, Bourguignon C, DiAngelis A, Hicks L, Sigurdsson A, Trope M, Tsukiboshi M, von Arx T (2007). Guidelines for the management of traumatic dental injuries II Avulsion of permanent teeth. Dent. Traumatol..

[3] Zhao YH, Zhang M, Liu NX, Lv X, Zhang J, Chen FM, Chen YJ (2013). The combined use of cell sheet fragments of periodontal ligament stem cells and platelet-rich fibrin granules for avulsed tooth reimplantation. Biomaterials.

[4] Goswami M, Chaitra TR, Chaudhary S, Manuja N, Sinha A (2011). Strategies for periodontal ligament cell viability: An overview. J. Conserv. Dent..

[5] Ripamonti U, Parak R, Petit JC (2009). Induction of cementogenesis and periodontal ligament regeneration by recombinant human transforming growth factor-beta3 in Matrigel with rectus abdominis responding cells. J. Periodontal Res..

[6] Buchmann R, Conrads G, Sculean A (2010). Short-term effects of systemic antibiotics during periodontal healing. Quintessence Int..

[7] Zhou Y, Li Y, Mao L, Peng H (2012). Periodontal healing by periodontal ligament cell sheets in a teeth replantation model. Arch. Oral Biol..

[8] Chen FM, Zhang J, Zhang M, An Y, Chen F, Wu ZF (2010). A review on endogenous regenerative technology in periodontal regenerative medicine. Biomaterials.

[9] Yang J, Yamato M, Kohno C, Nishimoto A, Sekine H, Fukai F, Okano T (2005). Cell sheet engineering: Recreating tissues without biodegradable scaffolds. Biomaterials.

[10] Reichert da Silva Assuncao L, Colenci R, Ferreira do-Amaral CC, Sonoda CK, Mogami Bomfim SR, Okamoto R, de Assis Golim M, Deffune E, Percinoto C, Penha de Oliveira SH (2011). Periodontal tissue engineering after tooth replantation. J. Periodontol..

[11] Dohan Ehrenfest DM, Rasmusson L, Albrektsson T (2009). Classification of platelet concentrates: From pure platelet-rich plasma (P-PRP) to leucocyte- and platelet-rich fibrin (L-PRF). Trends Biotechnol..

[12] Kassem M (2004). Mesenchymal stem cells: Biological characteristics and potential clinical applications. Cloning Stem Cells.

[13] Jiang Y, Jahagirdar BN, Reinhardt RL, Schwartz RE, Keene CD, Ortiz-Gonzalez XR, Reyes M, Lenvik T, Lund T, Blackstad M, Du J, Aldrich S, Lisberg A, Low WC, Largaespada DA, Verfaillie CM (2002). Pluripotency of mesenchymal stem cells derived from adult marrow. Nature.

[14] Menicanin D, Bartold PM, Zannettino AC, Gronthos S (2010). Identification of a common gene expression signature associated with immature clonal mesenchymal cell populations derived from bone marrow and dental tissues. Stem Cells Dev..

[15] Zhang J, Li ZG, Si YM, Chen B, Meng J (2014). The difference on the osteogenic differentiation between periodontal ligament stem cells and bone marrow mesenchymal stem cells under inflammatory microenviroments. Differentiation.

[16] Zhou J, Shi S, Shi Y, Xie H, Chen L, He Y, Guo W, Wen L, Jin Y (2011). Role of bone marrow-derived progenitor cells in the maintenance and regeneration of dental mesenchymal tissues. J. Cell Physiol..

[17] Ayatollahi M, Soleimani M, Geramizadeh B, Imanieh MH (2011). Insulin-like growth factor 1 (IGF-I) improves hepatic differentiation of human bone marrow-derived mesenchymal stem cells. Cell Biol. Int..

[18] Kramer PR, Nares S, Kramer SF, Grogan D, Kaiser M (2004). Mesenchymal stem cells acquire characteristics of cells in the periodontal ligament in-vitro. J. Dent. Res..

[19] Tsai CH, Shen SY, Zhao JH, Chang YC (2009). Platelet-rich fibrin modulates cell proliferation of human periodontally related cells in-vitro. J. Dent. Sci..

[20] Ji B, Sheng L, Chen G, Guo S, Xie L, Yang B, Guo W, Tian W (2015). The combination use of platelet-rich fibrin and treated dentin matrix for tooth root regeneration by cell homing. Tissue Eng. Part A.

[21] Ayatollahi M, Salmani MK, Geramizadeh B, Tabei SZ, Soleimani M, Sanati MH (2012). Conditions to improve expansion of human mesenchymal stem cells based on rat samples. World J. Stem Cells.

[22] Azarpira MR, Shahcheraghi GH, Ayatollahi M, Geramizadeh B (2015). Tissue engineering strategy using mesenchymal stem cell-based chitosan scafolds in growth plate surgery: A preliminary study in rabbits. Orthop. Traumatol. Surg. Res..

[23] Peng L, Jia Z, Yin X, Zhang X, Liu Y, Chen P, Ma K, Zhou C (2008). Comparative analysis of mesenchymal stem cells from bone marrow, cartilage, and adipose tissue. Stem Cells Dev..

[24] Lu W, Fu C, Song L, Yao Y, Zhang X, Chen Z, Li Y, Ma G, Shen C (2013). Exposure to supernatants of macrophages that phagocytized dead mesenchymal stem cells improves hypoxic cardiomyocytes survival. Int. J. Cardiol..

[25] Kawaguchi H, Hirachi A, Hasegawa N, Iwata T, Hamaguchi H, Shiba H, Takata T, Kato Y, Kurihara H (2004). Enhancement of periodontal tissue regeneration by transplantation of bone marrow mesenchymal stem cells. J. Periodontol..

[26] Li H, Yan F, Lei L, Li Y, Xiao Y (2009). Application of autologous cryopreserved bone marrow mesenchymal stem cells for periodontal regeneration in dogs. Cells Tissues Organs.

[27] Chen YL, Chen PKT, Jeng LB, Huang CS, Yang LC, Chung HY, Chang SCN (2008). Periodontal regeneration using ex-vivo autologous stem cells engineered to express the BMP-2 gene: An alternative to alveolaplasty. Gene Ther..

[28] Yang Y, Rossi FMV, Putnins EE (2010). Periodontal regeneration using engineered bone marrow mesenchymal stromal cells. Biomaterials.

[29] Simsek SB, Keles GC, Baris S, Cetinkaya BO (2012). Comparison of mesenchymal stem cells and autogenous cortical bone graft in the treatment of class II furcation defects in dogs. Clin. Oral Investig..

[30] Hasegawa M, Yamato M, Kikuchi A, Okano T, Ishikawa I (2005). Human periodontal ligament cell sheets can regenerate periodontal ligament tissue in an athymic rat model. Tissue Eng..

[31] Wu G, Cui Y, Ma L, Pan X, Wang X, Zhang B (2013). Repairing cartilage defects with bone marrow mesenchymal stem cells induced by CDMP and TGF-β1. Cell Tissue Bank..

[32] Ding G, Liu Y, Wang W, Wei F, Liu D, Fan Z, An Y, Zhang C, Wang S (2010). Allogeneic periodontal ligament stem cell therapy for periodontitis in swine. Stem Cells.

[33] Chen FM, Zhao YM, Jin Y, Shi S (2012). Prospects for translational regenerative medicine. Biotechnol. Adv..

[34] Feng F, Akiyama K, Liu Y, Yamaza T, Wang TM, Chen JH, Wang BB, Huang GTJ, Wang S, Shi S (2010). Utility of PDL progenitors for in-vivo tissue regeneration: A report of 3 cases. Oral Dis..

[35] Zeighami S, Hadjibabaie M, Ashouri A, Sarayani A, Khoee SH, Mousavi S, Radfar M, Ghavamzadeh A (2014). Assessment of cyclosporine serum concentrations on the incidence of acute graft versus host disease post hematopoietic stem cell transplantation. Iran. J. Pharm. Res..

[36] Mehdizadeh M, Hajifathali A, Tabarraee M, Tavakoli-Ardakani M (2013). Plerixafor in the treatment of stem cell mobilization failure; first experience in Iran. Iran. J. Pharm. Res..

[37] Dolgin E (2015). Next-generation stem cell therapy poised to enter EU market. Nat. Biotechnol..

